# Forest edges have high conservation value for bird communities in mosaic landscapes

**DOI:** 10.1002/ece3.2273

**Published:** 2016-06-28

**Authors:** Julien Terraube, Frédéric Archaux, Marc Deconchat, Inge van Halder, Hervé Jactel, Luc Barbaro

**Affiliations:** ^1^ Section of EcologyDepartment of BiologyUniversity of TurkuFIN‐20014TurkuFinland; ^2^ BiogecoINRAUniv. BordeauxF‐33610CestasFrance; ^3^ IrsteaUR EFNOF‐45290Nogent‐sur‐VernissonFrance; ^4^ DynaforINPTEI PurpanINRAUniv. ToulouseF‐31320AuzevilleFrance

**Keywords:** Biodiversity, community specialization index, conservation value index, ecosystem services, foraging guilds, fragmented forests, functional traits

## Abstract

A major conservation challenge in mosaic landscapes is to understand how trait‐specific responses to habitat edges affect bird communities, including potential cascading effects on bird functions providing ecosystem services to forests, such as pest control. Here, we examined how bird species richness, abundance and community composition varied from interior forest habitats and their edges into adjacent open habitats, within a multi‐regional sampling scheme. We further analyzed variations in Conservation Value Index (CVI), Community Specialization Index (CSI) and functional traits across the forest‐edge‐open habitat gradient. Bird species richness, total abundance and CVI were significantly higher at forest edges while CSI peaked at interior open habitats, i.e., furthest from forest edge. In addition, there were important variations in trait‐ and species‐specific responses to forest edges among bird communities. Positive responses to forest edges were found for several forest bird species with unfavorable conservation status. These species were in general insectivores, understorey gleaners, cavity nesters and long‐distance migrants, all traits that displayed higher abundance at forest edges than in forest interiors or adjacent open habitats. Furthermore, consistently with predictions, negative edge effects were recorded in some forest specialist birds and in most open‐habitat birds, showing increasing densities from edges to interior habitats. We thus suggest that increasing landscape‐scale habitat complexity would be beneficial to declining species living in mosaic landscapes combining small woodlands and open habitats. Edge effects between forests and adjacent open habitats may also favor bird functional guilds providing valuable ecosystem services to forests in longstanding fragmented landscapes.

## Introduction

Forest edges are widespread landscape elements in many European regions, due to a long history of forest fragmentation driven by agricultural and urbanization dynamics. The importance of forest edges for multi‐taxa biodiversity has been previously acknowledged, but the mechanisms underlying variations in the direction and magnitude of edge effects have been seldom explored (McCollin 1998; Ries et al. 2004; Ewers and Didham 2006a). The magnitude of edge effects may increase with the contrast between forest edge and adjacent open habitats and with the degree of landscape fragmentation, forest habitat area and cumulative effects of multiple edges (Fletcher 2005; Reino et al. 2009). Edge effects can have profound consequences for animal population dynamics (Fahrig 2003). Woodland bird nesting success is often reduced at edges by higher rates of nest predation and parasitism (Flaspohler et al. 2001; Chalfoun et al. 2002). Forest edges can also have negative effects on open‐habitat birds, as demonstrated in eucalypt plantations of Portuguese farmlands (Reino et al. 2009). As a consequence, edges have often been perceived by landscape ecologists or conservation planners as ecological traps, associated with the decline of forest habitat specialists in fragmented landscapes (Ries et al. 2004; Fletcher 2005; Laiolo and Rolando 2005).

However, habitat fragmentation and edge effects can also be perceived as positive ecological drivers (Fahrig 2003), for example by favoring early‐successional forest bird species with unfavorable conservation status in Europe and North‐America (Pons et al. 2003; Sanderson et al. 2006; Reino et al. 2009). By creating small‐scale variation in habitat heterogeneity, forest edges can enhance bird and insect diversity, particularly in large‐scale conifer plantations (Paquet et al. 2006; van Halder et al. 2011). Moreover, fragmentation and edge effects may also act as an environmental filter that determines species persistence via functional traits, potentially enhancing the local density of insectivorous birds (González‐Gómez et al. 2006; Barbaro et al. 2012) and thus increasing the capacity of bird assemblages to provide ecosystem services such as pest biocontrol (Jones et al. 2005; Whelan et al. 2015). Edge responses are often species‐ or guild‐specific, depending on habitat preferences and species traits (Balestrieri et al. 2015), and these attributes determine the observed patterns of variation in species abundance with distance from the forest edge (Ries et al. 2004; Ewers and Didham 2006a). Most edge‐related studies either do not actually consider distance from edge as a continuous factor, or concentrate on one side of the interior−exterior gradient only (usually from forest edge into its interior), rather than considering both sides of the forest edges (Ewers and Didham 2006b; Zurita et al. 2012). Moreover, as the type of landscape matrix surrounding forest fragments markedly influences edge effects, only multi‐site comparative studies can account for the variation in particular attributes of the landscape mosaic bordering forest fragments (Fletcher et al. 2007).

In such a context, there is a need for specifying the role of habitat edges between woodland patches and open areas in the spatial distribution of bird species diversity in mosaic landscapes combining forests fragments, seminatural grasslands and farmlands (González‐Gómez et al. 2006; Paquet et al. 2006; van Halder et al. 2011; Lindenmayer et al. 2015). Conservation implications of such studies are likely to be critical since recent studies have highlighted important population declines in forest specialist birds, including long‐distance migrant insectivores (Gregory et al. 2007; Vickery et al. 2014). These negative population trends could be related to change in woodland structure including reduced understorey vegetation or dead wood (Ouin et al. 2015). They could also result from change in woodland management leading to the alteration of breeding, wintering and stopover forest habitats (Rodewald and Brittingham 2004; Vickery et al. 2014).

Here, we investigated the response of bird communities to edges between forests and open habitats in a multi‐region sampling design in French temperate mosaic landscapes. We specifically analyzed bird species‐ and community‐level responses along the entire transition from forests to open habitats, in order to address: (1) how bird species richness and abundance, conservation value and community specialization vary in relation to distance from forest edge (i.e., from open habitat to interior forest habitat); and (2) how species‐specific edge responses differ among bird guilds.

## Materials and Methods

### Study areas

**Figure 1:**
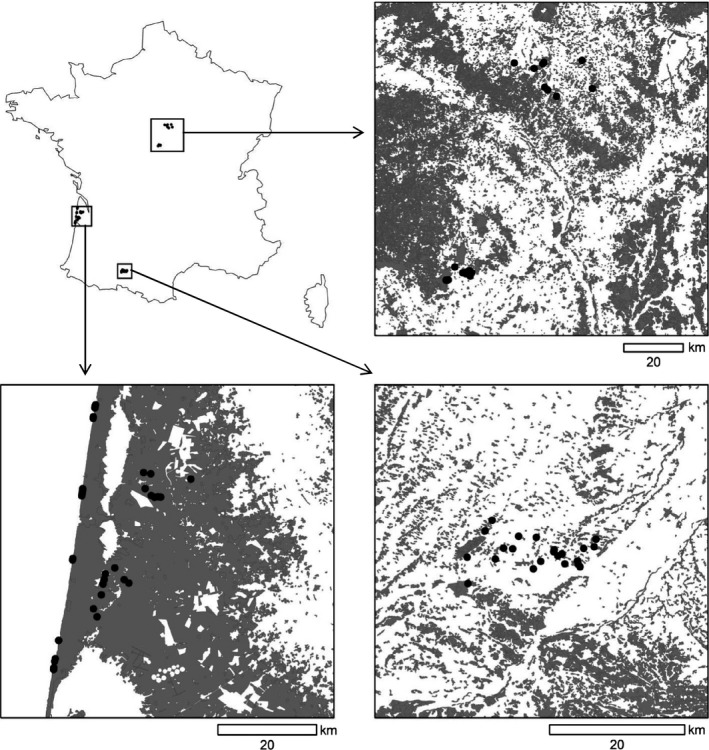
Location of the three study areas in Centre, Aquitaine and Midi‐Pyrénées regions. Boxes around the map represent enlarged views of each study area showing the location of sampling sites (black dots) in relation to forest cover (dark gray) and open habitats (white).

The study was conducted in three regions of France: two in the south‐west (Aquitaine and Midi‐Pyrénées) and one in central France (Centre‐Val de Loire) during spring of 2011 (see Fig. 1). In Aquitaine, almost one million hectares of maritime pines *Pinus pinaster* have been planted since the 19th century representing the largest plantation forest in Europe, where landscape is dominated by a mosaic of maritime pine plantations of different ages, clear‐cuts and herbaceous firebreaks. Firebreaks were created in order to minimize the risks associated to large fires, providing an interesting case to study the complex response of bird communities to sharp edges in a mosaic forest landscape. The climate is thermo‐Atlantic (mean annual temperature 12°C, mean annual rainfall 700 mm) and the elevation is low (c. 50 m a.s.l.). The study area in Midi‐Pyrénées (Haute‐Garonne district) is a temperate agro‐forested landscape, characterized by edges between broadleaved forest patches and agricultural land that present a sharp contrast as a result of regular management by farmers. Forest edges are quantitatively important because of the high number of small woodlots, dominated by oaks, *Quercus robur* and *Q. pubescens,* with total forest covering approximately 15% of total area. The region is hilly (250–400 m a.s.l.) and has a sub‐Atlantic climate with slight mountainous and Mediterranean influences (mean annual temperature 12.5°C; mean annual precipitation 750 mm). The study area in the Centre region encompassed two distinct subareas located ca. 75 km from each other, in the Loiret and Cher districts. The area is mostly dedicated to intensive crop (Loiret) and crop and apple production (Cher). Small deciduous forest fragments still persist on poorest soils, representing ca 18% of the total area. Most are oak‐hornbeam coppice‐with‐standards and used for timber and fuel wood production, presenting sharp edges with adjacent crop areas (see Appendix S1). Climate is sub‐Atlantic with some continental influence (mean annual temperature 11.3°C, mean annual precipitation 740 mm). The region is flat with mean elevation around 140 m (Loiret) and 225 m a.s.l. (Cher). Most of the forest edges in the Centre region were regularly managed with tractor‐mounted hedge trimmers to limit forest canopy growth over the fields. Trees in forest interiors are typically cut every 15–20 years.

Bird sampling was carried out on 80 sites as follows: 33 sites in Aquitaine, 26 sites in Midi‐Pyrénées and 21 sites in Centre region. In each region, we selected forest patches representative of the dominant forest habitat types. Degraded and small forest patches, with low, irregular forest cover were discarded because they were not representative of the forest stands present in the three regions. Edges too close to each other were also discarded to avoid multiple edge effects (as a potential source of bias, see Fletcher 2005). In each region, we selected half of forest patches adjacent to ploughed open habitats (oilseed rape fields in Midi‐Pyrénées and Centre and corn/buckwheat fields in Aquitaine) and the other half adjacent to unploughed open habitats (permanent grasslands in Midi‐Pyrénées and Aquitaine and apple orchards in Centre). In the Aquitaine region, study sites also included coastal pine forests adjacent to gray dune vegetation and interior pine forests adjacent to herbaceous firebreaks (ploughed or unploughed grasslands). There was no difference in habitat structure between coastal and interior pine plantations.

### Bird sampling

**Figure 2:**
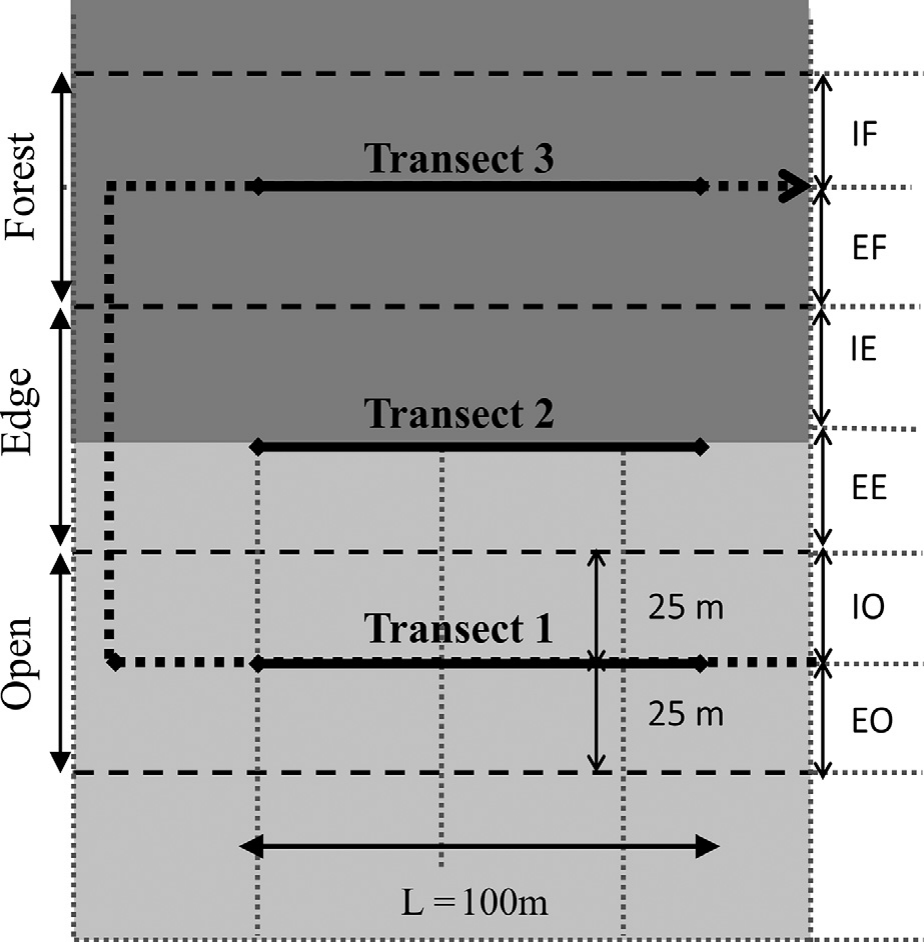
Bird sampling design used to analyze edge responses: three transects were simultaneously covered by three observers within three habitat types, forest (dark gray), edge and open habitat (light gray), defining six habitat strips along the forest‐edge‐open habitat gradient: Interior Forest (IF); Exterior Forest (EF); Interior (forest) Edge (IE), Exterior (open‐habitat) Edge (EE), Interior Open habitat (IO) and Exterior Open habitat (EO).

Bird communities were sampled in three 100 m‐long and 50 m‐wide linear transects parallel to the forest edge, within three habitat types (forests, edges and open habitats). The transects were divided into six 100 m‐long and 25 m‐wide habitat strips (interior forest, exterior forest, interior edge, exterior edge, interior open habitat and exterior open habitat, see Fig. 2). Linear transects were preferred over point counts because transects are less prone to bias due to bird movements and can be used for low bird densities (Bibby et al. 2000; Buckland 2006). Bird sampling was carried out by experienced observers walking along transects and locating, all birds seen or heard within the 25 m‐wide strips, except when clearly over‐flying the observer (migratory birds, raptors, swifts and swallows). Observers walked slowly enough to maximize the number of birds they encountered without risking double counting. Moving birds sighted while walking along the transect were given the location at first detection. Bird sampling was conducted twice (April and May) in the breeding season of 2011 (same period of time in the three regions), from 7:00 to 11:00 am, when birds are most active, and poor weather conditions were avoided (rain, strong wind or fog). For further data analysis, we used the maximum number of individuals recorded per species over the two visits.

We used a sampling design that allowed accounting for distance to the forest edge while minimizing the variation in species detectability within the edge. A distance of 25 m is actually considered as a distance allowing an optimal detectability (probability of detection by the observer close to 1) for most forest passerines (Brotons and Herrando 2003). Several studies on the depth of edge influence (DEI) have shown that edge effects do not penetrate further than 50 m for various taxa, including plants or insects (Harper and Macdonald 2001; Heliolä et al. 2001; Magura 2002; Harper et al. 2005; Roume et al. 2011 and references therein). Although the width of edge effect reported for birds can be deeper than 50 m, it rarely exceeds 70–80 m except for some interior specialist species or guilds that do not occur within our study sites located in longstanding fragmented temperate landscapes (Restrepo and Gómez 1998; Brand and George 2001; Fletcher 2005). Bird sampling was therefore designed according to previous studies that had demonstrated that 50 m was a recurrent threshold of approximate edge influence for both forest vegetation and birds, including avian foraging activity and insectivory levels (Baker et al. 2002; Harper et al. 2005; Rodewald and Vitz 2005; Barbaro et al. 2012; Bereczki et al. 2015). Moreover, this distance was compatible with the radius width of 25 m used for bird transect counts and allowed us to sample even small woodland patches and their edges.

### Bird community indices

A Conservation Value Index (CVI) was calculated for each habitat along the gradient from interior forests over edges to open habitats (Pons et al. 2003; Paquet et al. 2006). This index takes into account the European Conservation status or SPEC (Species of European Conservation Concern; Birdlife International 2004; see Appendix S2) and the log‐transformed abundance of the species contacted during the counts. The Conservation Value Index was calculated for each habitat on the forest‐edge‐open habitat gradient as follows (Pons et al. 2003; Paquet et al. 2006):

$${\text{CVI}}_{j}=\sum_{i=1}^{i=N_{j}}\log\left(a_{ij}+1\right)\times {\text{SPEC}}_{i},$$

where *N*_*j*_ is the total number of species recorded, *a*_*ij*_ the abundance of species *i* in the considered habitat strip *j*, and SPEC_*i*_ is the SPEC value of the species *i*.

For each transect, a Community Specialization Index (CSI) was calculated, reflecting the mean specialization level of species present in a given community (Julliard et al. 2006; Barnagaud et al. 2011). To calculate the CSI, we used the degree of habitat specialization for a given species (Species Specialization Index [SSI]), which was quantified as the coefficient of variation (SD/mean) of its densities across habitats (using the 18 habitat classes recorded by observers of the French breeding bird survey [FBBS] during point counts) (see Julliard et al. 2006 for more details). The CSI was then calculated for each community as the average specific specialization index of all individuals detected within a given strip. CSI in strip *j* was thus given by:

$${\text{CSI}}_{j}=\frac{\sum_{i=1}^{i=N_{j}}a_{ij}\left({\text{SSI}}_{i}\right)}{\sum_{i=1}^{i=N_{j}}a_{ij}},$$

where *N*_*j*_ is the total number of species recorded, *a*_*ij*_ the abundance of individuals of the species *i*, both in the habitat strip *j*, and SSI_*i*_ its specialization index (Devictor et al. 2008).

### Data analyses

Edge responses were analyzed with Poisson Generalized Linear Mixed Models (GLMM) to account for the lack of independence among bird counts between strips within the same site (Zuur et al. 2009). Sites were treated as random effects while region and habitat strip (i.e., a categorical variable with six modalities defined in the ‘Bird sampling design’ section) were defined as fixed effects. A two‐way interaction between these two variables was also included. We first analyzed variation in total species richness and abundance, CSI and CVI, in relation to distance from the forest interior to the exterior open habitat, in the 80 sites surveyed during this study. Second, we examined whether the edge response observed for each species also varied according to four major life‐history traits, expected to be good predictors of species response to fragmentation and of vulnerability to global change: adult diet, foraging method, migratory behavior and nest site selection (Barbaro and van Halder 2009; Vetter et al. 2011). Trait data were gathered from Cramp and Simmons (1980). Species were assigned to the three main food types that constitute the majority of adult diet during the breeding season: insectivorous species, mixed‐diet of insects and seeds, and granivorous species. Foraging methods were categorized as ground probers, ground gleaners, canopy gleaners, understorey gleaners, or bark foragers. Migratory behavior distinguished resident, short‐distance migrants, early long‐distance migrants and late long‐distance migrants, and nest site selection was categorized as species nesting in tree cavities, open nest on ground, open nest in shrub and open nest in tree (Barbaro and van Halder 2009).

Preliminary analyses using IndVal method (Dufrêne and Legendre 1997) were conducted to assess which of the commonest species had a higher degree of specificity and fidelity to the six habitat strips considered in this study. The 22 species, which had a maximal indicator value higher than 10%, were included in the analysis that aimed at assessing species‐specific response of birds to forest edges.

At the species level, many species had null abundance in unfavorable habitats (e.g., open habitats for a forest species). To avoid zero‐inflation of species‐specific models, we therefore analyzed the variation in each species abundance in four habitats rather than six: two corresponding to the specific‐species habitat preference and two corresponding to the adjacent habitat. For example, for forest species, we analyzed their abundance variation in the two forest strips (IF and EF, *d *=* *0 and 25 m, Fig. 2) and the two edge habitat strips (IE and EE, *d *=* *50 and 75 m, Fig. 2), while for edge species, we analyzed their abundance variation in the two edge strips and in one strip of the two other adjacent habitat types (exterior forest EF and interior open habitat IO strips, *d* = 25 and 100 m; Fig. 2). In each case, three different distance functions (see Reino et al. 2009) were tested by specifying linear (t_d1_ = *d*), logarithmic (t_d2_ = log_10_ [*d *+* *1]) and power (t_d3_ = *d* ^2^) transformations of the predictor variable, where *d* is the distance from forest interior (full model: species abundance, t_d (1,2,3)_+ region; interior forest habitat set as a base in all models). We chose to consider distance as a continuous factor as it better describes the extent and magnitude of edge effects for species (Ewers and Didham 2006b). We evaluated support for all models using AIC_*c*_ (AIC corrected for small sample size, Akaike 1973), the difference between the best model and other candidate models (ΔAIC_*c*_) and Akaike weights (*w*_i_), which represent relative support for each model in the set of three candidate models (‘Linear’, ‘Logarithmic’ and ‘Power’) and summed to one (Burnham and Anderson 2002).

## Results

### Bird community‐level response to forest edges

Altogether, 1891 individuals from 53 bird species were recorded along the 480 transects located across the three regions. Bird species communities were dominated by forest generalists, including chaffinch *Fringilla coelebs* (*n* = 257 individuals), great tit *Parus major* (*n* = 170), blackcap *Sylvia atricapilla* (*n* = 164) and chiffchaff *Phylloscopus collybita* (*n* = 150; Appendix S2). Mean total abundance and species richness were higher at forest edges than in other habitats, including interior forests, in Centre and Midi‐Pyrénées but not in Aquitaine where forest edges did not differ from forest interiors (GLMM, edge × region effect: *z* = −0.906; *P *=* *0.365 for abundance and *z* = −1.592; *P *=* *0.11 for richness).

**Figure 3:**
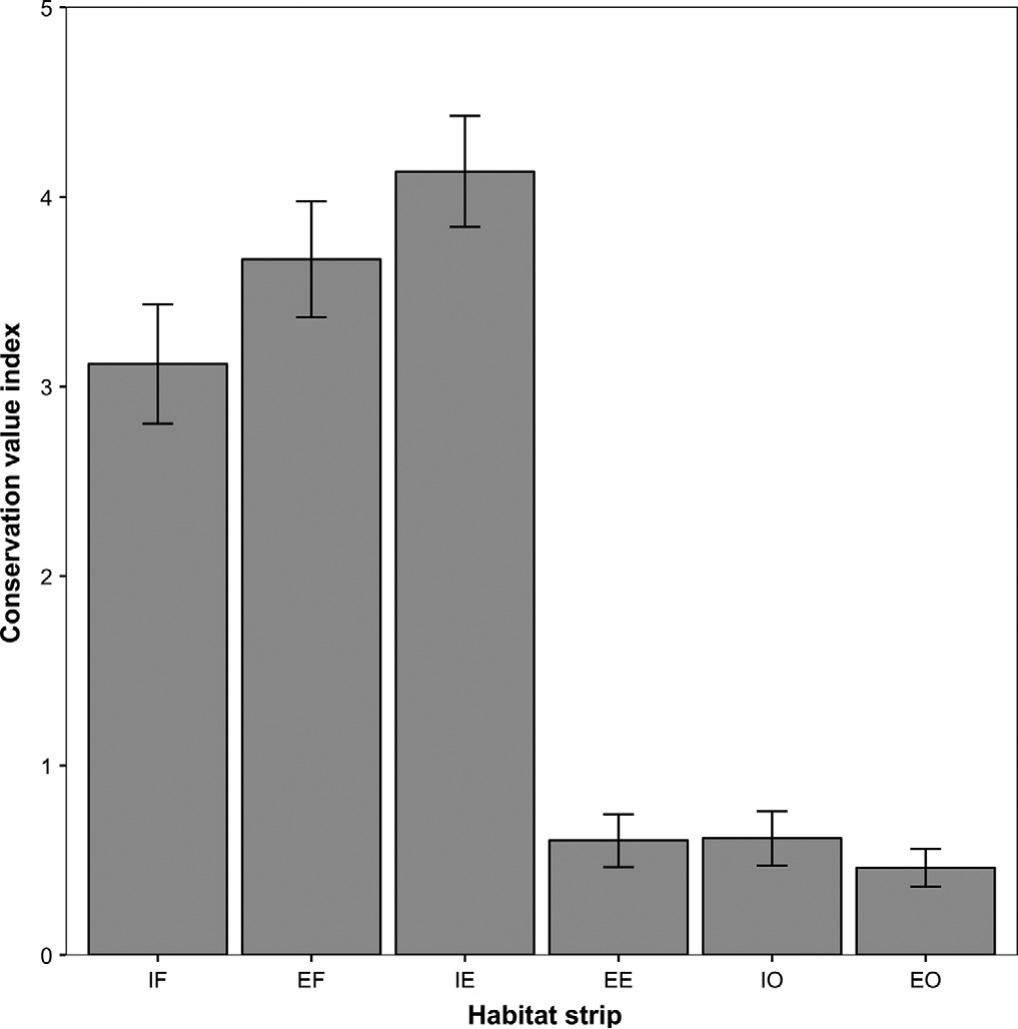
Changes in mean ± SE bird conservation value index (CVI) along the forest‐edge‐open habitat gradient: Interior Forest (IF); Exterior Forest (EF); Interior (forest) Edge (IE), Exterior (open‐habitat) Edge (EE), Interior Open habitat (IO), and Exterior Open habitat (EO).

**Figure 4:**
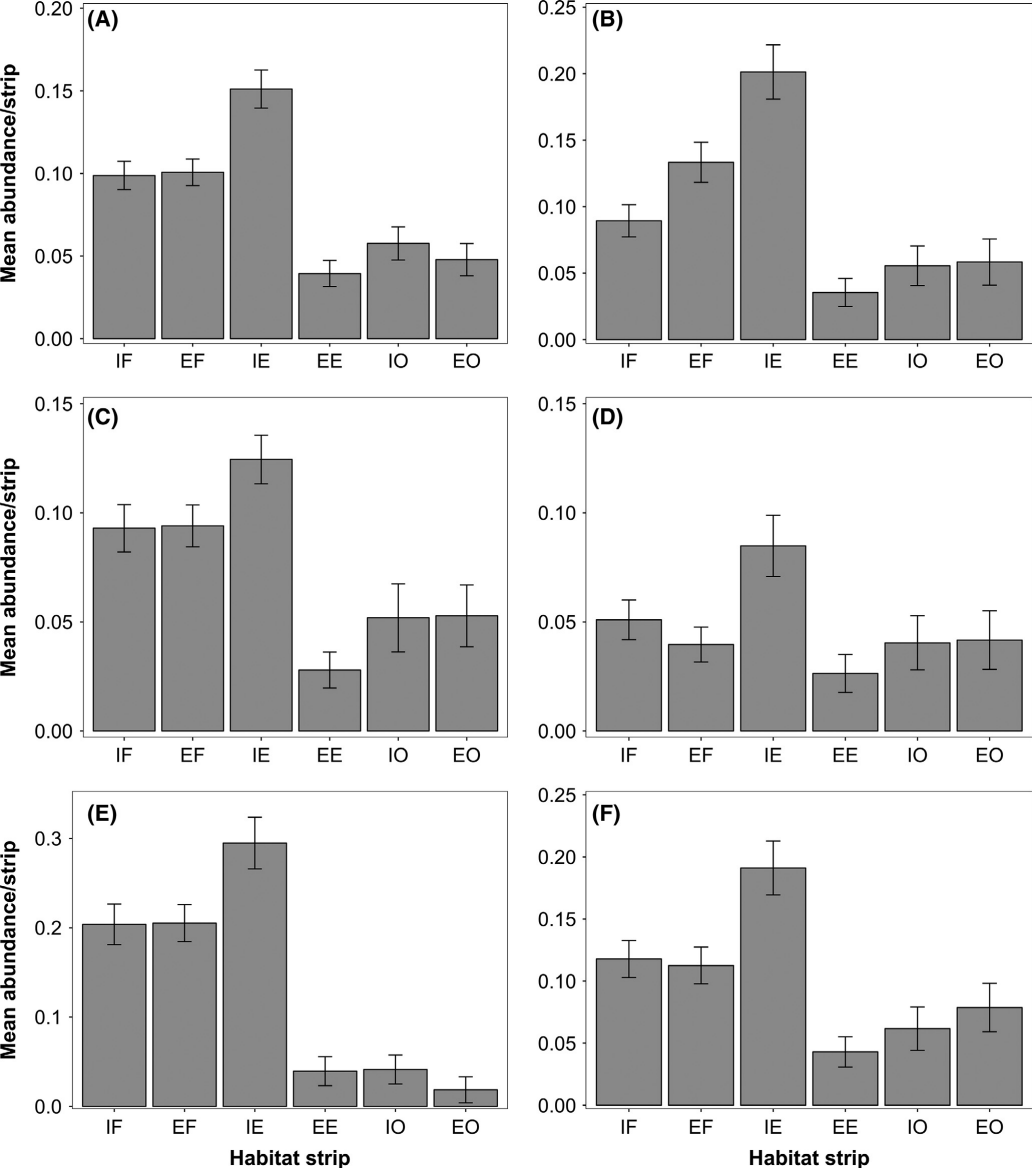
Mean abundance ± SE of insectivorous birds (A), understorey gleaning birds (B), resident birds (C), late long‐distance migratory birds (D), cavity‐nesting birds (E) and understorey‐nesting birds (F) along the forest‐edge‐open habitat gradient: Interior Forest (IF); Exterior Forest (EF); Interior (forest) Edge (IE), Exterior (open‐habitat) Edge (EE), Interior Open habitat (IO), and Exterior Open habitat (EO).

Mean bird Community Specialization Index continuously increased from 0.74 in the most interior forest transects to 1.29 in the most exterior open habitats and was significantly higher in interior and exterior open habitats than in edges and forests (GLMM, edge effect: *z* = 5.802; *P *<* *0.0001 and *z* = 7.032; *P *<* *0.0001, respectively). Mean bird Conservation Value Index increased along the gradient from interior forests to forest edges and then decreased to its lowest values in open habitats and their edges (Fig. 3). There was a significant interactive effect of edge and region on CVI as this index was significantly higher at interior edges than at interior forests and exterior edges in Midi‐Pyrénées (edge × region effect: *z* = 5.44; *P *<* *0.0001). For bird guilds based on adult diet and foraging methods (Fig. 4A and B), migratory behavior (Fig. 4C and D) and nest location (Fig. 4E and F), we found significantly higher abundances at interior edges than in interior forests and open habitats for insectivorous birds (GLMM, edge effect: *z* = 19.89; *P *<* *0.0001), resident species and long‐distance migrants (*z* = 3.764, *P *<* *0.0002 and *z* = 3.095; *P *<* *0.002), tree cavity nesters and birds nesting in open nests located in shrubs (*z* = 4.037; *P *<* *0.0001 and *z* = 3.033; *P *<* *0.005).

### Bird species‐level responses to edges

Two forest species, the long‐tailed tit *Aegithalos caudatus* and the blue tit *Cyanistes caeruleus*, showed a significant positive edge response with higher abundance in interior edges, and a pattern of exponential increase from interior forests to interior edges (Tables 1, 2). The abundance of six species increased in forest with increasing distance to forest edge. Woodpigeon *Columba palumbus*, chaffinch, wren *Troglodytes troglodytes*, chiffchaff, song thrush *Turdus philomelos* and common cuckoo *Cuculus canorus* were significantly more abundant in transects located in interior forests, while there was a similar but nonsignificant trend for turtle dove *Streptopelia turtur*, crested tit *Parus cristatus* and short‐toed treecreeper *Certhia brachydactyla*. Woodpigeon, song thrush, and common cuckoo abundance increased exponentially from interior edges to forest interior, whereas this increase was linear in the cases of chaffinch, wren and chiffchaff (Tables 1, 2). Six forest generalists showed no response to forest edges, namely great tit, European nuthatch *Sitta europaea*, robin *Erithacus rubecula*, blackcap, great‐spotted woodpecker *Dendrocopos major* and the western Bonelli's warbler *Phylloscopus Bonelli* (Tables 1, 2). Only the tree pipit *Anthus trivialis* can be considered a true edge species since its abundance increased exponentially with distance to forest edge and was maximal in exterior (open) edges and exterior open habitats. The abundance of two open‐habitat species, the stonechat *Saxicola torquata* and the tawny pipit *Anthus campestris*, increased also exponentially with distance from the edge, and was higher in the transects located in exterior open habitats (Tables 1, 2).

**Table 1:** Model selection results for three functions explaining variation in bird species abundance in relation to distance from forest edges (three transformations of the predictor variable *d*, distance from forest interior: (1) linear (t_d1_ = *d*); (2) power (t_d3_ = *d* ^2^); and (3) logarithmic (t_d2_ = log_10_ [*d *+* *1]))

Models	Linear				Power				Logarithmic			
Species abundance	*K*	AIC_*c*_	Δ_*i*_	*ω*_*i*_	*K*	AIC_*c*_	Δ_*i*_	*ω*_*i*_	*K*	AIC_*c*_	Δ_*i*_	*ω*_*i*_
Woodpigeon	4	160.98	0.32	0.32	4	160.85	0.20	0.34	4	**160.82**	**0.16**	**0.34**
Turtle dove	4	96.48	0.16	**0.34**	4	96.48	0.17	0.34	4	96.53	0.22	0.33
Common cuckoo	4	79.59	0.92	0.27	4	79.18	0.51	0.33	4	78.83	0.16	**0.40**
Great‐spotted woodpecker	4	158.27	0.16	**0.39**	4	158.64	0.53	0.33	4	158.92	0.81	0.28
Woodlark	4	49.15	0.96	0.27	4	48.76	0.57	0.33	4	48.36	0.16	**0.40**
Tree pipit	4	117.49	1.41	0.23	4	116.83	0.75	0.32	4	116.21	0.14	**0.44**
Tawny pipit	4	39.54	1.11	0.26	4	39.05	0.62	0.33	4	38.60	0.16	**0.41**
Wren	4	207.71	0.16	**0.53**	4	208.84	1.30	0.17	4	209.91	2.37	0.30
Robin	4	189.25	0.31	0.33	4	189.34	0.40	0.32	4	189.11	0.16	**0.35**
Stonechat	4	43.54	0.71	0.29	4	43.26	0.43	0.33	4	42.99	0.16	**0.38**
Song thrush	4	79.00	0.56	0.30	4	78.77	0.33	0.34	4	78.60	0.16	**0.37**
Blackcap	4	205.40	0.16	**0.38**	4	205.81	0.57	0.31	4	205.89	0.65	0.30
Whitethroat	4	64.26	0.27	0.33	4	64.21	0.21	0.33	4	64.16	0.16	**0.34**
Western Bonelli's warbler	4	110.18	0.16	**0.41**	4	110.65	0.63	0.32	4	111.01	0.99	0.27
Chiffchaff	4	232.25	0.16	**0.65**	4	234.25	2.16	0.24	4	235.81	3.72	0.11
Long‐tailed tit	4	106.63	1.83	0.20	4	105.66	0.85	0.33	4	104.94	0.14	**0.47**
Crested tit	4	168.49	0.16	**0.50**	4	169.50	1.17	0.30	4	170.34	2.01	0.20
Blue tit	4	215.79	1.53	0.22	4	215.01	0.74	0.69	4	214.40	0.14	**0.45**
Great tit	4	314.37	0.84	0.28	4	314.06	0.53	0.33	4	313.66	0.14	**0.40**
European nuthatch	4	100.09	0.58	0.30	4	99.84	0.34	0.33	4	99.64	0.14	**0.37**
Short‐toed treecreeper	4	206.87	0.16	**0.48**	4	207.79	1.08	0.31	4	208.55	1.85	0.21
Chaffinch	4	198.76	0.16	**0.59**	4	200.31	1.72	0.27	4	201.73	3.13	0.13

This table includes the number of predictors (*K*), the Akaike information criterion score (AIC_*c*_), the difference between the given model and the most parsimonious model (∆_*i*_) and Akaike weight (*ω*_*ι*_). The most parsimonious model is highlighted in bold (see Methods for more detailed description of the procedure).

**Table 2:** Parameter estimates from the best models in Table 1 (highlighted in bold) explaining variation in species‐specific bird abundance in relation to distance from forest interior strip

Species abundance	*β*	SE	*z*‐value	Pr(*β *> *z*)
**Woodpigeon**	−**2.675**	**0.782**	−**3.419**	**<0.001**
Turtle dove	−0.017	0.011	−1.666	0.096
**Common cuckoo**	−**2.777**	**1.289**	−**2.154**	**0.031**
Great‐spotted woodpecker	−0.007	0.006	−1.096	0.273
Woodlark	5.027	2.783	1.806	0.070
**Tree pipit**	**3.424**	**1.274**	**2.686**	**0.007**
**Tawny pipit**	**14.990**	**4.879**	**3.071**	**0.002**
**Wren**	−**0.020**	**0.010**	−**3.250**	**<0.001**
Robin	0.265	0.557	0.475	0.634
**Stonechat**	**16.480**	**7.644**	**2.156**	**0.031**
**Song thrush**	−**3.246**	**1.322**	−**2.454**	**0.014**
Blackcap	−0.002	0.003	−0.700	0.483
Whitethroat	0.011	0.008	1.348	0.177
Western Bonelli's warbler	0.009	0.005	−1.011	0.31
**Chiffchaff**	−**0.008**	**0.003**	−**2.449**	**0.014**
**Long‐tailed tit**	**3.535**	**1.322**	**2.673**	**0.007**
Crested tit	−0.008	0.004	−1.894	0.058
**Blue tit**	**1.660**	**0.671**	**2.472**	**0.013**
Great tit	0.376	0.422	0.892	0.372
European nuthatch	1.005	1.019	0.985	0.324
Short‐toed treecreeper	−0.010	0.005	−1.908	0.056
**Chaffinch**	−**0.008**	**0.002**	−**3.066**	**0.002**

## Discussion

Our study pointed out a large diversity of bird species and guild responses to edges, even among species or guilds with the same general habitat requirements (i.e., forest or open‐habitat species). We actually found different types of species responses along the habitat gradient at both sides of forest edges. Forest edges appeared as important habitats in terms of conservation value, as a consequence of higher total abundance and species richness in bird assemblages using this habitat as well as higher occurrence of sensitive species exhibiting particular traits (insectivore and cavity‐nesting species). These results support the hypothesis of higher availability of resources in forest‐open habitat edges (Ries et al. 2004; Laiolo and Rolando 2005; Riffell et al. 2015). Forest edges should thus be considered keystone habitats, providing bird‐related ecosystem services (predation) in mosaic landscapes (González‐Gómez et al. 2006; Zamora et al. 2010; Barbaro et al. 2012; Bereczki et al. 2015).

Both habitat structure and floristic composition are important to explain the distribution of birds across forest‐open habitat edges. Bird communities appear to respond to a complex of forest habitat attributes, including growth stage and the structure and composition of understorey vegetation (Hewson et al. 2011). As a consequence, higher abundance of breeding birds in forest edges could be linked to differences in small‐scale vegetation composition and structure affecting prey abundance and foraging efficiency (Van Wilgenburg et al. 2001; González‐Gómez et al. 2006). Ouin et al. (2015), studying the same forest edges in the Midi‐Pyrénées site, showed that forest edges support a higher density of tree microhabitats (TMH thereafter) than forest interior, particularly because of the presence of larger trees at forest edges. They also confirmed that some TMH types were more abundant in forest edges, such as bark loss patches, cracks, sap runs, and epiphytes. These TMH are known to provide suitable habitat for many species (Bouget et al. 2014). Forest edges, with higher TMH density and higher vegetation complexity (e.g., higher shrub cover and higher richness and diversity of vascular plants; Alignier et al. 2014) are likely providing birds with more foraging and nest sites (Ouin et al. 2015).

Our results thus support the hypothesis that forest edges are important habitats for breeding birds in longstanding man‐made landscapes. Indeed, bird species richness and total abundance were higher at interior forest edges than at any other habitat type, although there was high inter‐regional variation. Moreover, species with less favorable conservation status had higher abundance at forest edges, as suggested by higher values of conservation index in this habitat. Additionally, the positive effect of forest edges on bird species richness and total abundance mainly resulted from the addition of species from both sides of the edge (and particularly from forest habitats) as very few species were true edge‐specialists in this study (see Imbeau et al. 2003). Even if results were statistically significant for only two species (blue and long‐tailed tits), two other forest species − great tit and nuthatch − followed this pattern of increased abundance at forest edges. These species are characterized by an insectivorous diet and nest located either in tree cavities or within shrubs, further supporting the role of higher habitat complexity in shaping local bird distribution (Brotons and Herrando 2003; Balestrieri et al. 2015). Higher prey availability and more abundant tree cavities in more complexly structured forest edges than forest interiors, are factors potentially explaining the observed pattern (Van Wilgenburg et al. 2001; Laiolo and Rolando 2005; Ouin et al. 2015), as well as the noticeable increase in avian insectivory observed in manipulative experiments (Barbaro et al. 2012; Bereczki et al. 2015).

In our study, there was an important variation in the magnitude of edge effect between regions. The positive edge effect on abundance and species richness was observed in two regions, in Midi‐Pyrénées and Centre, but this was not the case in the Aquitaine region. This pattern is probably linked to inter‐regional variation in forest composition, edge contrast, and adjacent habitat management. In Aquitaine, forest stands were composed of homogeneous pine plantations with sharp edges, i.e., limited presence of broadleaved understorey (van Halder et al. 2011). This homogeneous structure in pine plantations resulted in low contrast between interior edges and interior forests, probably explaining the lack of positive bird responses to edges in this region (Ries et al. 2004). The lower abundance of bird species showing a positive response to forest edges in coniferous forests compared to broadleaved stands could also explain this pattern (Barbaro and van Halder 2009). Actually, forests in the two other regions were principally composed of broadleaved species, less intensively managed and with softer edges (Roume et al. 2011), which could explain their higher selection by foraging forest birds (Brotons and Herrando 2003; Bereczki et al. 2015).

Our results also suggest a possible negative edge effect for six bird species since the abundance of these species was higher in interior forests (Flaspohler et al. 2001; Ewers and Didham 2006a). In this study, woodpigeon, common cuckoo, and song thrush were all in this case. Interestingly, these three species are typically associated with heterogeneous landscapes mixing open habitats and woodlands (Inglis et al. 1994; Bellamy et al. 2003; Peach et al. 2004; Paquet et al. 2006). These species are thus likely examples of habitat complementation (Dunning et al. 1992), with birds selecting interior forest habitats for breeding and adjacent open habitats to forests for foraging, like for example the hoopoe *Upupa epops* or the mistle thrush *Turdus viscivorus* (Barbaro and van Halder 2009).

The absence of interior forest specialist species in our dataset, such as the middle spotted woodpecker (*Dendrocops medius*), may be related to the small size of the selected forest patches, particularly in Midi‐Pyrénées and Centre regions but may also reflect an edge avoidance extending beyond 75 m (the maximum distance in our case) in the most sensitive birds. Therefore, it would be valuable to repeat this study in other regions enabling the selection of larger forest patches in order to decipher how long‐distance edge effects influence sensitive forest species in forest landscapes with less anthropogenic footprint.

Forest edge avoidance in grassland species has been documented in other European farmland regions (Reino et al. 2009) resulting from increased nest predation near edges due to changes in vegetation structure affecting nest conspicuousness and predator foraging behavior (Chalfoun et al. 2002). Our results highlighted that open‐habitat communities were the most specialized in terms of habitat (Devictor et al. 2010). This suggests that open‐habitat species could be negatively affected by the fragmentation of open habitats by forested patches in mosaic landscapes (Archaux and Martin 2009). Particularly, two species restricted to open habitats (stonechat and tawny pipit) showed negative responses to forest edges in terms of abundance. Forest edges can also be associated with higher predation risks for adult birds, and the negative selection of open habitats near edges by conspicuous singing birds during the breeding period could be explained by this spatial variation in predation risk. Forest edge avoidance could alternatively result from the evolutionary history of grassland birds in meadows and steppes with virtually no trees, which resulted in a strong aversion to less familiar features (Fletcher 2005; Rodewald and Vitz 2005; Reino et al. 2009). This could be the case for the tawny pipit, a typical open habitat specialist. By contrast, the stonechat is often associated to shrubland areas interspersed with trees, and this species has been showed to positively respond to forest edges in Portuguese farmland (Reino et al. 2009).

### Implications for conservation

Globally, bird response to edge was either species‐ or guild‐specific but our results provided evidence that forest edges in fragmented landscapes can be important habitats for generalist forest birds as well as for some specialist edge and interior forest species (Imbeau et al. 2003). However, further research is required to investigate whether higher bird densities (corresponding to individual birds singing) at edges are also paralleled by higher fitness parameters (breeding success, survival) inducing spatial variation in population dynamics. For example, higher nest predation rates at edges could result in lower breeding success for songbirds, as already shown in other studies (Flaspohler et al. 2001). Our results confirmed that forest edges are valuable for conserving and even enhancing biodiversity in managed, fragmented landscapes, by increasing local habitat heterogeneity and mitigating the effects of landscape homogenization linked to modern forestry practices (Dolman et al. 2007). Combined with negative factors in African wintering areas for migratory birds, changes in temperate forest structure and composition are thought to be responsible for the decline in common woodland birds in Europe (Gregory et al. 2007; Vickery et al. 2014). Moreover, forest edges are not only important habitats for breeding birds but they are also extensively used by migratory passerine birds during stopovers (Keller et al. 2009). Refuelling in high‐quality stopover stations is critical for migratory birds, as low body condition during this phase of the annual cycle can increase mortality (Rodewald and Brittingham 2004). Higher avian predation rates on invertebrates have been actually recorded experimentally at forest edges in fragmented landscapes, suggesting that prey availability and accessibility may be much higher at edges compared to forest interiors (González‐Gómez et al. 2006; Barbaro et al. 2012; Bereczki et al. 2015). The loss of favorable stopover sites linked to current land‐use changes in European landscapes has been proposed as one of the factors related to the long‐term population decline in Afro‐Palearctic migrant birds (Sanderson et al. 2006; Gregory et al. 2007; Vickery et al. 2014), pointing out the conservation value of forest edge habitats for stopover insectivore migrants. A better integration between forestry practices and optimal management of forest edges is thus needed, including complex understorey structures at forest edges, a measure potentially highly beneficial to a large range of forest bird species but also to various insect taxa such as butterflies (van Halder et al. 2011). Finally, our results also provide evidence that seminatural open habitats are important habitats for some specialist species with high conservation value in our study sites. Because some of these open habitat species exhibited negative responses to forest edges, landscape planning should aim at increasing the area of open patches in forested landscapes. This may provide both habitat for the persistence of these species and corridors enhancing dispersal toward extensive intact open habitats, particularly in continuous forest plantations.

## Conflict of Interest

None declared.

## Supporting information


**Appendix S1.** Land cover percentages for 10 main land‐cover types in a buffer of 500 m around each sampling site included in this study.

**Appendix S2.** Mean transect abundance and total abundance of 50 most abundant bird species recorded across the three regions.
