# Supplementary material for: Forest edges have high conservation value for bird communities in mosaic landscapes
Source: Ecol Evol. 2016 Jun 28;6(15):5178–89. doi: 10.1002/ece3.2273 (PMC4984496; doi:10.1002/ece3.2273)
Supplement: Supplementary file 1 — Appendix S1. Land cover percentages for 10 main land‐cover types in a buffer of 500 m around each sampling site included in this study. [file ECE3-6-5178-s001.docx]

| **Site** | **Region** | **Forests** | **Young pine plantations** | **Clear-cuts/Young plantations^1^** | **Open areas with sparse trees** | **Orchards** | **Grasslands** | **Dunes** | **Fallows** | **Agricultural fields** | **Others^2^** |
| --- | --- | --- | --- | --- | --- | --- | --- | --- | --- | --- | --- |
| A1 | Aquitaine | 84.2 | 0.0 | 0.8 | 3.7 | 0.0 | 7.2 | 0.0 | 0.0 | 0.0 | 4.1 |
| A2 | Aquitaine | 82.7 | 0.0 | 3.6 | 0.0 | 0.0 | 8.5 | 0.0 | 0.0 | 0.0 | 5.2 |
| A3 | Aquitaine | 82.1 | 0.0 | 0.0 | 0.0 | 0.0 | 11.0 | 0.0 | 0.0 | 0.0 | 6.9 |
| A4 | Aquitaine | 83.9 | 0.0 | 0.1 | 0.0 | 0.0 | 10.4 | 0.0 | 0.0 | 0.0 | 5.5 |
| A5 | Aquitaine | 60.9 | 5.7 | 7.4 | 11.7 | 0.0 | 10.3 | 0.0 | 0.0 | 0.0 | 3.9 |
| A6 | Aquitaine | 33.5 | 6.6 | 44.2 | 2.5 | 0.0 | 7.1 | 0.0 | 0.0 | 0.0 | 6.1 |
| A7 | Aquitaine | 28.5 | 3.8 | 33.5 | 28.1 | 0.0 | 2.7 | 0.0 | 0.0 | 0.0 | 3.4 |
| A8 | Aquitaine | 27.5 | 13.3 | 34.6 | 14.2 | 0.0 | 0.4 | 0.0 | 0.0 | 6.0 | 3.9 |
| A9 | Aquitaine | 76.3 | 4.1 | 9.3 | 0.0 | 0.0 | 6.9 | 0.0 | 0.0 | 0.0 | 3.3 |
| A10 | Aquitaine | 49.4 | 21.6 | 22.1 | 0.0 | 0.0 | 4.4 | 0.0 | 0.0 | 0.0 | 2.5 |
| A11 | Aquitaine | 62.9 | 2.3 | 19.5 | 0.1 | 0.0 | 12.0 | 0.0 | 0.0 | 0.0 | 3.2 |
| A12 | Aquitaine | 83.3 | 0.0 | 10.6 | 0.0 | 0.0 | 3.6 | 0.0 | 0.0 | 0.0 | 2.5 |
| A13 | Aquitaine | 90.4 | 0.0 | 0.0 | 0.0 | 0.0 | 0.0 | 0.0 | 0.2 | 5.0 | 4.4 |
| A14 | Aquitaine | 41.9 | 0.0 | 48.3 | 2.4 | 0.0 | 0.0 | 0.0 | 0.0 | 5.3 | 2.1 |
| A15 | Aquitaine | 68.1 | 0.0 | 16.5 | 4.5 | 0.0 | 0.0 | 0.0 | 0.0 | 6.0 | 4.8 |
| A16 | Aquitaine | 81.5 | 0.0 | 2.6 | 1.9 | 0.0 | 0.0 | 0.0 | 0.0 | 5.3 | 8.3 |
| A17 | Aquitaine | 82.2 | 0.5 | 8.0 | 3.3 | 0.0 | 0.0 | 0.0 | 0.0 | 3.7 | 2.3 |
| A18 | Aquitaine | 41.5 | 0.8 | 3.9 | 19.4 | 0.0 | 0.0 | 30.9 | 0.0 | 0.0 | 3.5 |
| A19 | Aquitaine | 38.6 | 8.5 | 0.0 | 15.6 | 0.0 | 0.0 | 32.0 | 0.0 | 0.0 | 5.2 |
| A20 | Aquitaine | 42.2 | 0.0 | 7.1 | 5.7 | 0.0 | 0.0 | 29.0 | 0.0 | 0.0 | 15.9 |
| A21 | Aquitaine | 37.0 | 3.9 | 8.8 | 9.6 | 0.0 | 0.0 | 28.0 | 0.0 | 0.0 | 12.8 |
| A22 | Aquitaine | 47.5 | 0.0 | 0.0 | 6.8 | 0.0 | 0.0 | 31.7 | 0.0 | 0.0 | 14.0 |
| A23 | Aquitaine | 47.9 | 0.0 | 0.0 | 4.8 | 0.0 | 0.0 | 23.2 | 0.0 | 0.0 | 24.1 |
| A24 | Aquitaine | 50.6 | 0.0 | 0.0 | 5.5 | 0.0 | 0.0 | 21.9 | 0.0 | 0.0 | 22.0 |
| A25 | Aquitaine | 36.0 | 0.6 | 10.7 | 3.6 | 0.0 | 0.0 | 18.2 | 0.0 | 0.0 | 30.9 |
| A26 | Aquitaine | 42.6 | 4.6 | 2.7 | 4.3 | 0.0 | 0.0 | 19.7 | 0.0 | 0.0 | 26.0 |
| A27 | Aquitaine | 41.0 | 9.4 | 0.0 | 3.7 | 0.0 | 0.0 | 21.2 | 0.0 | 0.0 | 24.8 |
| A28 | Aquitaine | 39.6 | 1.9 | 6.9 | 3.1 | 0.0 | 0.0 | 20.1 | 0.0 | 0.0 | 28.4 |
| A29 | Aquitaine | 35.7 | 0.0 | 12.4 | 1.3 | 0.0 | 0.0 | 14.9 | 0.0 | 0.0 | 35.7 |
| A30 | Aquitaine | 42.2 | 0.0 | 5.7 | 1.5 | 0.0 | 0.0 | 15.3 | 0.0 | 0.0 | 35.4 |
| A31 | Aquitaine | 39.8 | 0.0 | 10.8 | 3.3 | 0.0 | 0.0 | 12.6 | 0.0 | 0.0 | 33.5 |
| A32 | Aquitaine | 45.3 | 0.0 | 4.4 | 3.3 | 0.0 | 0.0 | 13.4 | 0.0 | 0.0 | 33.6 |
| A33 | Aquitaine | 49.7 | 0.0 | 2.0 | 2.3 | 0.0 | 0.0 | 14.5 | 0.0 | 0.0 | 31.5 |
| CV1 | Centre | 25.3 | 0.0 | 0.0 | 0.0 | 9.3 | 14.7 | 0.0 | 0.0 | 49.2 | 1.6 |
| CV2 | Centre | 54.9 | 0.0 | 0.0 | 0.0 | 7.2 | 27.5 | 0.0 | 0.0 | 10.1 | 0.4 |
| CV3 | Centre | 65.4 | 0.0 | 0.0 | 0.0 | 6.6 | 25.9 | 0.0 | 0.0 | 0.7 | 1.3 |
| CV4 | Centre | 7 .1 | 0.0 | 0.0 | 0.0 | 12.3 | 6.6 | 0.0 | 0.0 | 71.0 | 3.0 |
| CV5 | Centre | 17.9 | 0.0 | 0.0 | 0.0 | 14 .9 | 11.2 | 0.0 | 0.0 | 54.7 | 1.2 |
| CV6 | Centre | 19.6 | 0.0 | 0.0 | 0.0 | 17.2 | 16.7 | 0.0 | 0.0 | 43.1 | 3.5 |
| CV7 | Centre | 13.8 | 0.0 | 0.0 | 0.0 | 9.1 | 32.9 | 0.0 | 0.0 | 36.9 | 7.3 |
| CV8 | Centre | 13.1 | 0.0 | 0.0 | 0.0 | 12.7 | 24.6 | 0.0 | 0.0 | 44.6 | 5.0 |
| CV9 | Centre | 30.3 | 0.0 | 0.0 | 0.0 | 4.3 | 26.3 | 0.0 | 0.0 | 26.3 | 12.7 |
| CV10 | Centre | 28.6 | 0.0 | 0.0 | 0.0 | 13.4 | 41.6 | 0.0 | 0.0 | 10.4 | 0.0 |
| CV11 | Centre | 80.2 | 0.0 | 0.0 | 0.0 | 11.6 | 5.6 | 0.0 | 0.0 | 0.0 | 3.1 |
| CC1 | Centre | 7.8 | 0.0 | 0.0 | 0.0 | 0.0 | 9.8 | 0.0 | 0.0 | 78.0 | 4.5 |
| CC2 | Centre | 31.2 | 0.0 | 0.0 | 0.0 | 0.0 | 4.3 | 0.0 | 0.0 | 59.1 | 5.5 |
| CC3 | Centre | 34.6 | 0.0 | 0.0 | 0.0 | 0.0 | 16.4 | 0.0 | 0.0 | 48.5 | 0.6 |
| CC4 | Centre | 25.8 | 0.0 | 0.0 | 0.0 | 0.0 | 10.4 | 0.0 | 0.0 | 63.8 | 0.0 |
| CC5 | Centre | 32.5 | 0.0 | 0.0 | 0.0 | 0.0 | 15.3 | 0.0 | 0.0 | 52.2 | 0.0 |
| CC6 | Centre | 42.3 | 0.0 | 0.0 | 0.0 | 0.0 | 3.6 | 0.0 | 0.0 | 52.3 | 1.9 |
| CC7 | Centre | 21.7 | 0.0 | 0.0 | 0.0 | 0.0 | 3.6 | 0.0 | 0.0 | 72.7 | 3.8 |
| CC8 | Centre | 16 .2 | 0.0 | 0.0 | 0.0 | 0.0 | 4.9 | 0.0 | 0.0 | 77.4 | 1.5 |
| CC9 | Centre | 31.6 | 0.0 | 0.0 | 0.0 | 0.0 | 9.7 | 0.0 | 0.0 | 54.5 | 4.2 |
| CC10 | Centre | 31.2 | 0.0 | 0.0 | 0.0 | 0.0 | 4.3 | 0.0 | 0.0 | 59.1 | 5.5 |
| C1 | Midi-Pyr | 13.5 | 0.0 | 0.0 | 0.0 | 0.0 | 29.5 | 0.0 | 0.0 | 48.9 | 8.1 |
| C2 | Midi-Pyr | 14.0 | 0.0 | 0.0 | 0.0 | 0.0 | 16.3 | 0.0 | 2.1 | 65.0 | 2.7 |
| C3 | Midi-Pyr | 16.5 | 0.0 | 0.0 | 0.0 | 0.0 | 9.3 | 0.0 | 0.0 | 70.2 | 4.0 |
| C5 | Midi-Pyr | 22.4 | 0.0 | 0.0 | 0.0 | 0.0 | 29.0 | 0.0 | 1.8 | 41.7 | 5.1 |
| C6 | Midi-Pyr | 36.6 | 0.0 | 0.0 | 0.0 | 0.0 | 2.7 | 0.0 | 0.0 | 56.8 | 3.9 |
| C7 | Midi-Pyr | 26.7 | 0.0 | 0.0 | 0.0 | 0.0 | 41.6 | 0.0 | 0.0 | 29.3 | 2.4 |
| C8 | Midi-Pyr | 21.9 | 0.0 | 0.0 | 0.0 | 0.0 | 14.4 | 0.0 | 0.5 | 41.8 | 21.5 |
| C9 | Midi-Pyr | 31.7 | 0.0 | 0.0 | 0.0 | 0.0 | 22.6 | 0.0 | 3.5 | 36.0 | 6.1 |
| P1 | Midi-Pyr | 38.6 | 0.0 | 0.0 | 0.0 | 0.0 | 42.1 | 0.0 | 0.4 | 13.7 | 5.1 |
| P2 | Midi-Pyr | 49.3 | 0.0 | 0.0 | 0.0 | 0.0 | 42.2 | 0.0 | 2.5 | 2.6 | 3.4 |
| P3 | Midi-Pyr | 20.9 | 0.0 | 0.0 | 0.0 | 0.0 | 26.7 | 0.0 | 0.0 | 46.2 | 6.2 |
| P4 | Midi-Pyr | 22.7 | 0.0 | 0.0 | 0.0 | 0.0 | 48.0 | 0.0 | 4.0 | 21.3 | 4.0 |
| P5 | Midi-Pyr | 10.2 | 0.0 | 0.0 | 0.0 | 0.0 | 23.4 | 0.0 | 0.3 | 63.4 | 2.7 |
| P7 | Midi-Pyr | 16.2 | 0.0 | 0.0 | 0.0 | 0.0 | 57.0 | 0.0 | 6.1 | 16.7 | 4.0 |
| P9 | Midi-Pyr | 7.5 | 0.0 | 0.0 | 0.0 | 0.0 | 12.9 | 0.0 | 2.5 | 69.7 | 7.4 |
| P10 | Midi-Pyr | 24.7 | 0.0 | 0.0 | 0.0 | 0.0 | 45.4 | 0.0 | 0.0 | 24.1 | 5.8 |
| P12 | Midi-Pyr | 26.0 | 0.0 | 0.0 | 0.0 | 0.0 | 37.4 | 0.0 | 1.8 | 31.2 | 3.7 |
| P13 | Midi-Pyr | 55.2 | 0.0 | 0.0 | 0.0 | 0.0 | 5.8 | 0.0 | 0.4 | 37.3 | 1.3 |
| P14 | Midi-Pyr | 45.9 | 0.0 | 0.0 | 0.0 | 0.0 | 28.3 | 0.0 | 1.0 | 20.7 | 4.1 |
| P15 | Midi-Pyr | 30.1 | 0.0 | 0.0 | 0.0 | 0.0 | 30.5 | 0.0 | 0.4 | 22.8 | 16.2 |
| P16 | Midi-Pyr | 34.4 | 0.0 | 0.0 | 0.0 | 0.0 | 37.7 | 0.0 | 0.5 | 22.0 | 5.3 |
| P17 | Midi-Pyr | 17.3 | 0.0 | 0.0 | 0.0 | 0.0 | 26.7 | 0.0 | 0.0 | 48.7 | 7.3 |

**Appendix A** : Land cover percentages for 10 main land-cover types in a buffer of 500m around each sampling site included in this study.

^1^ Tree height<3m.

^2^ The category « Others » includes the ocean for sites located along the dunes in Aquitaine region.
