# Supplementary material for: Forest edges have high conservation value for bird communities in mosaic landscapes
Source: Ecol Evol. 2016 Jun 28;6(15):5178–89. doi: 10.1002/ece3.2273 (PMC4984496; doi:10.1002/ece3.2273)
Supplement: Supplementary file 2 — Appendix S2. Mean transect abundance and total abundance of 50 most abundant bird species recorded across the three regions. [file ECE3-6-5178-s002.docx]

| **Common name** | **Latin name** | **Abbr.** | **SPEC status** | **Internal forests** | **External forests** | **Internal edges** | **External edges** | **Internal open** | **External open** | **Total abundance** |
| --- | --- | --- | --- | --- | --- | --- | --- | --- | --- | --- |
| Long-tailed tit | *Aegitalos caudatus* | aegcau | Non-SPEC | 0.05 | 0.03 | 0.23 | 0.06 | 0.00 | 0.00 | 25 |
| Skylark | *Alauda arvensis* | alaarv | SPEC-3 | 0 | 0 | 0 | 0 | 0.11 | 0.042 | 4 |
| Tawny pipit | *Anthus campestris* | antcam | SPEC-3 | 0 | 0 | 0 | 0 | 0.19 | 0.25 | 11 |
| Tree pipit | *Anthus trivialis* | anttri | Non-SPEC | 0.06 | 0.05 | 0.17 | 0.42 | 0.30 | 0.13 | 45 |
| Common linnet | *Carduelis cannabina* | carcan | SPEC-2 | 0 | 0 | 0.01 | 0.03 | 0.04 | 0.04 | 4 |
| European goldfinch | *Carduelis carduelis* | carcar | Non-SPEC | 0 | 0 | 0.05 | 0.00 | 0.04 | 0.04 | 6 |
| Euroepan greenfinch | *Carduelis chloris* | carchl | SPEC-4 | 0.09 | 0.11 | 0.17 | 0.00 | 0.00 | 0.04 | 28 |
| Siskin | *Carduelis spinus* | carspi | Non-SPEC | 0 | 0.02 | 0 | 0 | 0 | 0 | 1 |
| Short-toed treecreeper | *Certhya brachydactyla* | cerbra | SPEC-4 | 0.31 | 0.27 | 0.28 | 0.00 | 0.00 | 0.00 | 63 |
| Hawfinch | *Coccothraustes coccothraustes* | cococc | Non-SPEC | 0.01 | 0.00 | 0.04 | 0.00 | 0.00 | 0.00 | 4 |
| Wood pigeon | *Columba palumbus* | colpal | SPEC-4 | 0.12 | 0.32 | 0.15 | 0.00 | 0.04 | 0.00 | 42 |
| Carrion crow | *Corvus corone* | corcor | Non-SPEC | 0.04 | 0.09 | 0.04 | 0.00 | 0.04 | 0.17 | 17 |
| Common cukoo | *Cuculus canorus* | cuccan | Non-SPEC | 0.03 | 0.12 | 0.04 | 0.03 | 0 | 0 | 14 |
| Blue tit | *Cyanistes caerulescens* | cyacae | SPEC-4 | 0.24 | 0.15 | 0.44 | 0.19 | 0.07 | 0.00 | 70 |
| Great spotted woodpecker | *Dendrocops major* | denmaj | Non-SPEC | 0.17 | 0.17 | 0.21 | 0.00 | 0.07 | 0.00 | 42 |
| Lesser spotted woodpecker | *Dendrocops minor* | denmin | Non-SPEC | 0.03 | 0.00 | 0.00 | 0.00 | 0.00 | 0.00 | 2 |
| Cirl bunting | *Emberiza cirlus* | embcir | SPEC-4 | 0.00 | 0.02 | 0.07 | 0.06 | 0.07 | 0.04 | 11 |
| Yellowhammer | *Emberiza citrinella* | embcit | SPEC-4 | 0 | 0 | 0.03 | 0.00 | 0.04 | 0.13 | 6 |
| European robin | *Erithacus rubecula* | erirub | SPEC-4 | 0.46 | 0.20 | 0.40 | 0.03 | 0.07 | 0.08 | 84 |
| Chaffinch | *fringilla coelebs* | fricoe | SPEC-4 | 0.87 | 0.94 | 0.81 | 0.29 | 0.04 | 0.08 | 203 |
| Crested lark | *Galerida cristata* | galcri | SPEC-3 | 0 | 0 | 0 | 0 | 0.07 | 0.08 | 4 |
| Eurasian jay | *Garrulus glandarius* | gargla | Non-SPEC | 0.09 | 0.06 | 0.13 | 0.06 | 0.00 | 0.00 | 23 |
| Melodious warbler | *Hippolais polyglotta* | hippol | SPEC-4 | 0 | 0.02 | 0.07 | 0.03 | 0.07 | 0.13 | 12 |
| Wood lark | *Lullula arborea* | lularb | SPEC-2 | 0 | 0 | 0.04 | 0.10 | 0.15 | 0 | 10 |
| Nightingale | *Luscinia megarhyncos* | lusmeg | SPEC-4 | 0.03 | 0.08 | 0.15 | 0.00 | 0.04 | 0.00 | 19 |
| White wagtail | *Motacilla alba* | motalb | Non-SPEC | 0.01 | 0.00 | 0.01 | 0.00 | 0.15 | 0.04 | 7 |
| Spotted flycatcher | *Muscicapa striata* | musstr | SPEC-3 | 0.01 | 0.02 | 0.03 | 0 | 0 | 0 | 4 |
| Northern weathear | *Oenanthe oenanthe* | oenoen | SPEC-3 | 0 | 0 | 0 | 0 | 0.04 | 0.04 | 2 |
| Eurasian golden oriole | *Oriolus oriolus* | oriori | Non-SPEC | 0.03 | 0.08 | 0.04 | 0 | 0 | 0 | 10 |
| Crested tit | *Parus cristatus* | parcri | SPEC-2 | 0.41 | 0.36 | 0.35 | 0.10 | 0 | 0 | 85 |
| Great tit | *Parus major* | parmaj | Non-SPEC | 0.45 | 0.47 | 0.96 | 0.06 | 0.19 | 0.17 | 149 |
| Black redstart | *Phoenicurus ochruros* | phooch | Non-SPEC | 0 | 0 | 0 | 0.03 | 0 | 0 | 1 |
| Common redstart | *Phoenicurus phoenicurus* | phopho | SPEC-2 | 0 | 0.02 | 0 | 0 | 0 | 0 | 1 |
| Bonelli's warbler | *Phylloscopus bonelli* | phybon | SPEC-2 | 0.14 | 0.09 | 0.11 | 0 | 0 | 0 | 25 |
| Common chiffchaff | *Phylloscopus collybita* | phycol | Non-SPEC | 0.65 | 0.48 | 0.57 | 0.00 | 0.04 | 0 | 127 |
| Willow warbler | *Phylloscopus trochilus* | phytro | Non-SPEC | 0.03 | 0.00 | 0.01 | 0 | 0 | 0 | 3 |
| Green woodpecker | *Picus viridis* | picvir | SPEC-2 | 0.05 | 0.02 | 0.05 | 0 | 0 | 0 | 9 |
| Dunnock | *Prunella modularis* | prumod | SPEC-4 | 0 | 0 | 0.04 | 0 | 0 | 0 | 3 |
| Firecrest | *Regulus ignicapilla* | regign | SPEC-4 | 0.03 | 0.06 | 0.04 | 0 | 0 | 0 | 9 |
| Stonechat | *Saxicola torquata* | saxtor | Non-SPEC | 0 | 0 | 0 | 0 | 0.11 | 0.21 | 8 |
| European nuthatch | *Sitta europaea* | siteur | Non-SPEC | 0.08 | 0.08 | 0.21 | 0 | 0 | 0 | 27 |
| Collared dove | *Streptotelia decaocto* | strdec | Non-SPEC | 0.01 | 0 | 0 | 0 | 0 | 0 | 1 |
| European turtle dove | *Streptotelia turtur* | strtur | SPEC-2 | 0.06 | 0.14 | 0.07 | 0.03 | 0.04 | 0 | 21 |
| Common starling | *Sturnus vulgaris* | stuvul | SPEC-3 | 0.09 | 0.03 | 0.05 | 0.00 | 0.19 | 0.04 | 19 |
| Blackcap | *Sylvia atricapilla* | sylatr | SPEC-4 | 0.59 | 0.41 | 0.73 | 0.00 | 0.04 | 0 | 129 |
| Whitethroat | *Sylvia communis* | sylcom | SPEC-4 | 0.01 | 0.00 | 0.07 | 0.16 | 0.15 | 0.25 | 21 |
| Wren | *Troglodytes troglodytes* | trotro | Non-SPEC | 0.33 | 0.45 | 0.31 | 0.00 | 0.00 | 0.04 | 80 |
| Blackbird | *Turdus merula* | turmer | SPEC-4 | 0.21 | 0.36 | 0.37 | 0.06 | 0.04 | 0 | 71 |
| Song thrush | *Turdus philomelos* | turphi | SPEC-4 | 0.04 | 0.14 | 0.05 | 0.00 | 0.00 | 0.04 | 17 |
| Mistle thrush | *Turdus viscivorus* | turvis | SPEC-4 | 0.08 | 0.12 | 0.12 | 0.10 | 0.04 | 0.17 | 31 |
| Hoopoe | *Upupa epops* | upuepo | SPEC-3 | 0.01 | 0.03 | 0.01 | 0.00 | 0.00 | 0.04 | 5 |

**Appendix B**. Mean transect abundance and total abundance of 50 most abundant bird species recorded across the three regions. SPEC status is indicated (Birdlife International 2004).
